# Cyclone Nargis and Myanmar: A wake up call

**DOI:** 10.4103/0974-2700.50745

**Published:** 2009

**Authors:** Fatimah Lateef

**Affiliations:** Director of Training and Education, Department of Emergency Medicine, Singapore General Hospital, Singapore

**Keywords:** Cyclone, disaster preparedness, rebuilding and reconstruction, tsunami

## Abstract

In early May 2008, Cyclone Nargis (CN) tore across the southern coastal regions of Myanmar, pushing a tidal surge through villages and rice paddies. The almost 12 foot wall of water and wind speed of over 200 km/hr killed tens of thousands of people and left hundreds of thousands homeless and vulnerable to injury and disease. Out of the 7.35 million living in the affected townships of Labutta, Bogale, Pyinsalu, Yangon, and many more, approximately 2.4 million were affected. Overall, more than 50 townships were affected by this most devastating cyclone in Asia since 1991. The Delta region, Myanmar's Rice Bowl, was severely damaged. The low-lying villages were submerged. There was widespread destruction of homes, critical infrastructure of the villages, roads, ferries, water, fuel, and electricity supplies. Our team from Singapore (called Team Singapore) reached out to at least 10 different villages during the time we were there. We ran mobile clinics daily at several locations and these operated from warehouses, temples, schools or any make shift buildings. The journey to the remote villages may take between 1 and 2 hours by road or by boat. We also ran mobile clinics at the township hospital, the rural healthcare centers, and an orphanage.

## INTRODUCTION

Besides the many benefits of the ocean, population living in coastal regions is at risk for meteorological and seismic hazards that originate from the seas. Tropical cyclones and tsunamis represent the most powerful and destructive of all marine hazards. Disasters such as these can expose the vulnerabilities in the capabilities of healthcare and community infrastructure to respond to them.[1,2]

## CYCLONE NARGIS

In early May 2008, Cyclone Nargis (CN) tore across the southern coastal regions of Myanmar, pushing a tidal surge through villages and rice paddies. The almost 12 foot wall of water and wind speed of over 200 km/hr killed tens of thousands of people and left hundreds of thousands homeless and vulnerable to injury and disease.

**Figure 1:**
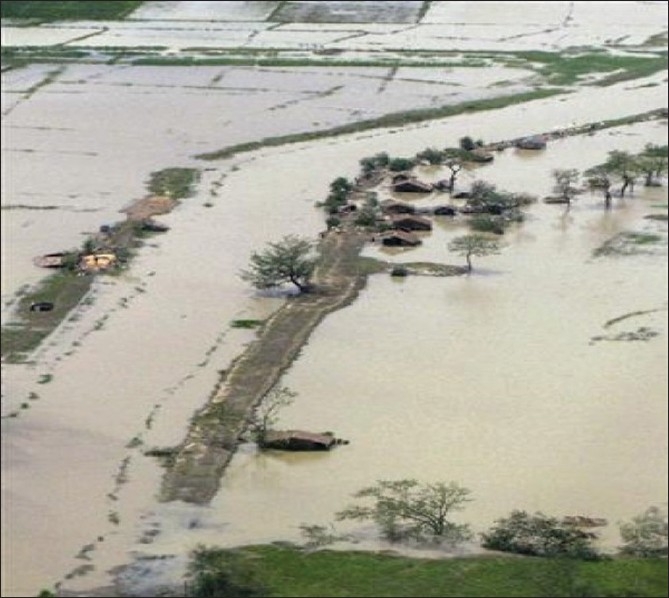
Aerial view of the submerged and destroyed Irrawady Delta

**Figure 2:**
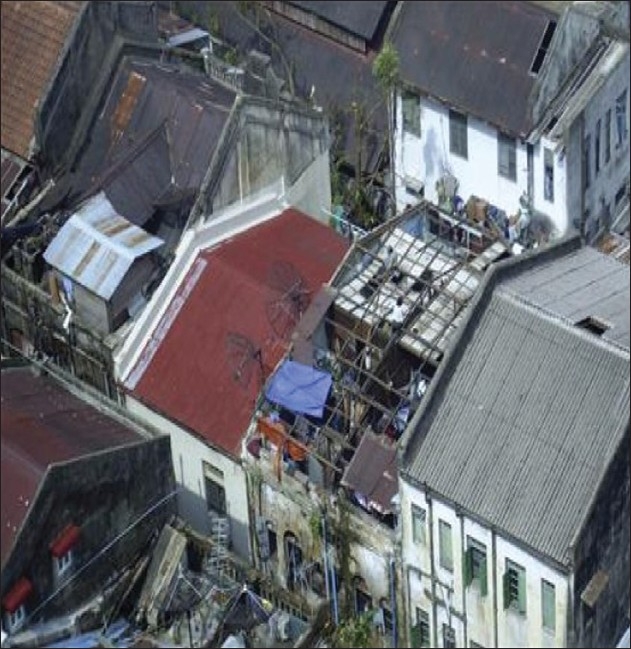
Destroyed buildings with roofs blown off (urban areas)

Out of the 7.35 million living in the affected townships of Labutta, Bogale, Pyinsalu, Yangon and many more, approximately 2.4 million were affected. Overall, more than 50 townships were affected by this most devastating cyclone in Asia since 1991. The Delta region, Myanmar's Rice Bowl, was severely damaged [Figure 1]. The low-lying villages were submerged. There was widespread destruction of homes, critical infrastructure of the villages, roads, ferries, water, fuel, and electricity supplies [Figure 2]. The people in the Delta region were mostly farmers, laborers, fishermen or traders. Their communities were cohesive despite being from different ethnic origins (there are 134 different ethnic groups in Myanmar).[3,4]

The Delta region is exposed to disasters like floods, tsunamis, cyclones as well as fire hazards. CN is one of the most controversial disasters, bringing forth issues of:[4,5]

- preparedness of a nation to handle a large scale crisis
- basic human rights issue
- community empowerment
- definition of basic healthcare needs of a nation and
- issues of environmental education

## HEALTHCARE AND MYANMAR

Besides the disaster, social and political are also the other issues Myanmar is facing. More than 50 years of internal conflict have led many citizens to forced migration. It is estimated that there are about 1 million people who are internally displaced and more than 1 million Myanmese refugees. About 155,000 of these refugees live in 9 official camps at the Thai-Myanmar border.[3,4]

Despite the fact stated that 2.2% of Myanmar GDP was spent on healthcare, basic healthcare services were virtually nonexistent in the villagers and rural areas. Where available, they were rudimentary and access to the majority of the population was limited. The general level of healthcare awareness was very low and public health education was lacking. They generally have no access to clean drinking water and there is little sanitation services in the rural area.[6,7]

The State Peace and Development Council spend less than US$1 (i.e. 60 cents) per person per year, on healthcare. Approximately, 1 in 4 households live below the poverty line. There are important disparities between the rural and urban areas and 70% of the population resides in the former. Many in the villages, including pregnant women and children, have substandard diet. Across Myanmar, about 40% of children fewer than 5 years have stunted growth and about 30% are underweight. There was a general lack of medication and if available, there was often sharing and splitting of these medications with others, making prescribed treatment ineffective. The use of traditional medicine is also very widespread and forms an integral part of the country's health services.

Malaria is the leading cause of morbidity and mortality with over 538,000 cases and 1,647 deaths reported in 2006. Dengue and dengue haemorrhagic fever cases have seasonal epidemics. Myanmar is also among the 22 countries with the highest burden of tuberculosis. An estimated 1.6% of the population is infected annually.

Pregnancy related deaths represent the leading cause of mortality amongst women of reproductive age group. The maternal mortality ranges from 230 to 580 per 100,000 live births.[3,4,6,7]

For every 1,000 population, there are currently 0.36 doctors, 0.2 nurses, 0.79 midwives, and 0.99 general healthworker. The corresponding figures for USA would be, 2.56 doctors, 9.37 nurses, 1.63 midwives and 20.96 healthcare worker per 1,000 citizens.[3]

A National Health Plan 2006-2011 has been developed to improve healthcare, response and fairness in the distribution of financial contributions. This national strategic plan has been formulated for reproductive health, child health, adolescent health, HIV/AIDS, tuberculosis and malaria control, water supply, sanitation and hygiene. Generally, World Health Organisation (WHO), UNICEF (United Nation Children's Fund), and UNDP (United nations Development Programme) contribute to many health related activities and programmes in Myanmar.[3,6]

## CONSTITUTING AND DISPATCHING TEAM SINGAPORE

Following the disaster, Team Singapore was formed. The team comprised of members/volunteers from different local nongovernmental organizations (NGOs such as The Singapore Red Cross and Mercy Relief) and healthcare institutions. The choice and numbers of personnel was decided based on updates and ground reports, media reports and other verified feedback received, pertaining to possible injuries, number of casualties, expected procedures and diseases, among other considerations. As these NGOs and institutions usually have their own standard operating procedures (SOP) and logistics, equipment and drug listings, these have to be aligned for Team Singapore, in the response to CN.

The team also had discussions and worked with many other local groups who wanted to make their representations felt and contribute in terms of manpower, money or other resources. For example, interpreters, which is an essential service required by the team, came from immigrants or expatriats from Myanmar who worked in Singapore. It is also mandatory to establish good and reliable ground contact with local hosts in Myanmar, as this will facilitate entry, transfer of equipment and aid, as well as other documentary arrangements. This could be in the form of a local NGO in the receiving country or other local associations. Communications and keeping up to date with organizations such as World Food Programme, the United Nations and UNHCR (United Nations High Commissioner for Refugee) too is important.

Vaccination for team members, psychological preparedness counseling and briefing by Foreign Affairs staff were compulsory. Packing of drugs and equipments, to ensure sufficient stock to function for up to a week was also done. The plan was to procure the rest of the drugs and equipment in Yangon. Cargo and air freight arrangements as well as local transportation in Myanmar were secured on a preliminary basis.

All set to go, member's spirits were indeed high, wanting to impact a change in the lives of those affected.

## ARRIVAL OF TEAM SINGAPORE IN MYANMAR

On arrival in Yangon, there were discussions with the local authorities. The Prime Minister's office in Yangon took charge of the National Natural Disaster Preparedness Coordinating Committee which was set up in response to CN. This committee was chaired by the Prime Minister himself. Various subcommittees were also formed by the local government to oversee various areas pertaining to the post disaster period. These committees were to plan a comprehensive programme for reconstruction and this was to comprise of the following:[8]

1. Transition Phase management. This was defined as the efforts required between the rescue and rehabilitation phase.
2. Short-term rebuilding. This was defined as the rapid improvement which could be made in both the urban and rural areas to have farmers and fishermen resume their livelihood as soon as possible.
3. Longer term rebuilding. This was to cover reconstruction and resettlement.
4. Preparedness and prevention. Here, it was suggested that the local authorities studied and looked into some form of early warning system, mechanism for mobilization, construction of stronger facilities and infrastructure, storm resistant roads and embankments, storm shelters and regeneration of the mangrove forests.

## TEAM SINGAPORE RESPONDS TO TWANTE TOWNSHIP

**Figure 3:**
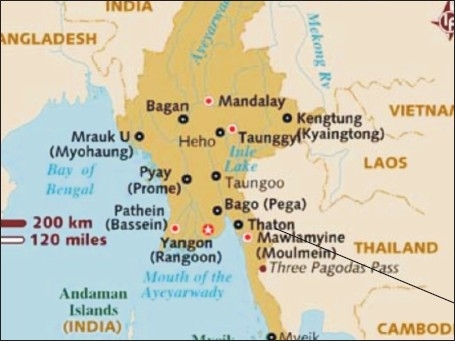
Yangon Division

Team Singapore was allocated the Twante Township [Figure 3], just to the east of the Yangon River, to work at. This is a township with a population of 251,709, with a rural predominance. From the local statistics given to the team, there was an estimated 31,463 children under 5 years in this area. There was only one township hospital with 25 beds and 5-6 rural health centers. There were also smaller sub-health centers where school health and maternal health services operated from. The area also had between 5 and 10 private clinics.

As in any disasters, the priority for the medical team was to manage the primary casualties (those with direct effect and injuries from CN) as well as the secondary casualties (e.g. those with anxiety or depression, those who witnessed destruction and death as well as family members of primary victims and the bereaved).

In the early phase, needs of the victims were highest for basic survival commodities as well as basic healthcare needs. The former would comprise of food, water, shelter and blankets whilst the latter involve front and first line care as well as more definitive care which can be provided by international or local humanitarian teams at field clinics, hospitals or more definitive institutions, if these had been preserved.

Team Singapore faced a unique situation in the work to be carried out. Unlike other large scale disasters where multiple local and international NGOs and organizations work together at Ground Zero, often coordinated by the United Nations. In Myanmar, each team was designated a specific locality/township to work in and given a local liaison officer, who helped to facilitate and coordinate the teams activities. He may also act as the interpreter.

## RESPONSE STATISTICS

**Figure 4:**
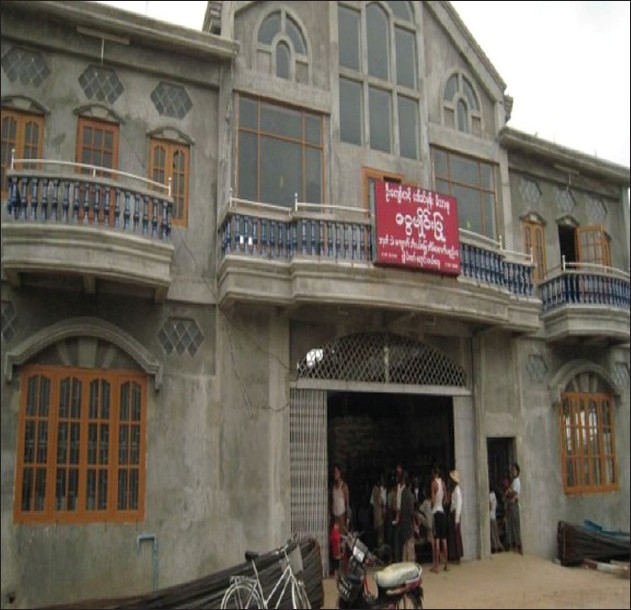
Preserved warehouse building where a mobile clinic was operated

**Figure 5:**
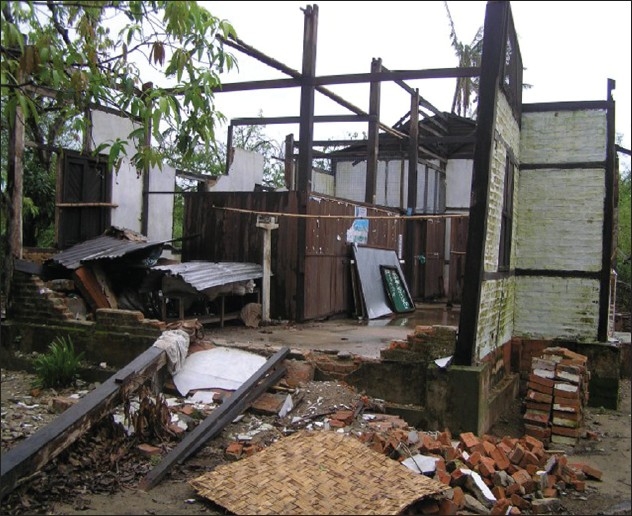
A destroyed village health center

**Figure 6:**
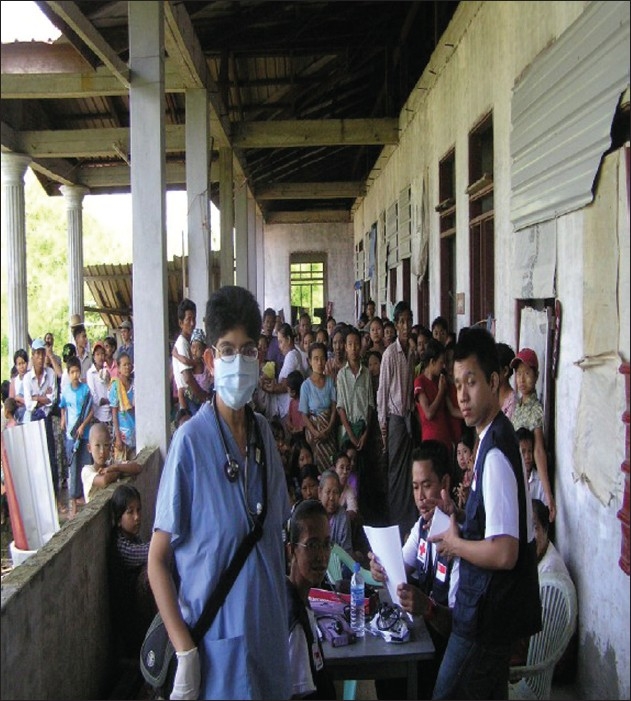
The patient waiting area outside an unfinished clinic building in a remote village where Team Singapore ran mobile clinics

**Figure 7:**
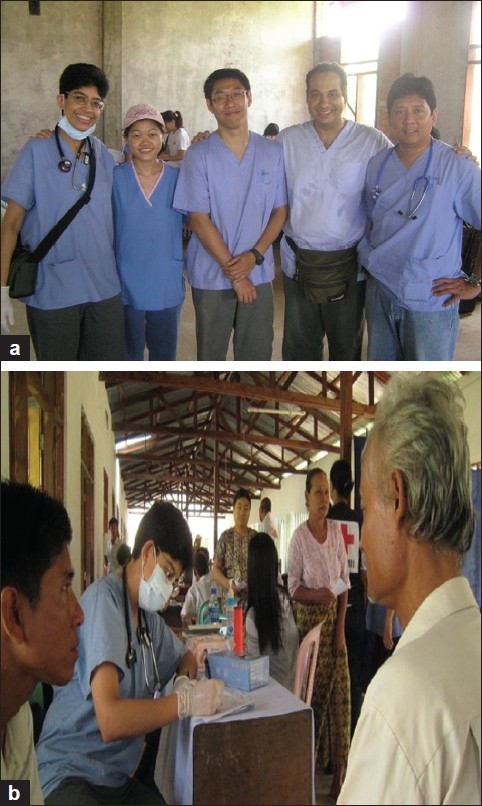
(a) Team Singapore doctors (b) Consultation makes shift area in a partially preserved village school

Team Singapore reached out to at least 10 different villages/rural areas during the time we were there. We ran mobile clinics daily at several locations and these operated from warehouses, temples, schools or any make shift buildings [Figures 4–7]. The journey to the remote villages may take between 1 and 2 hours by road or by boat.

We also ran mobile clinics at the township hospital, the rural healthcare centers and an orphanage.

For the 9 operational days, Team Singapore treated a total of 4,710 patients [Table 1]. Adults made up 62% (2,921) whilst paediatric patients (≤ 12 years) comprised 38% of those treated. The commonest diagnoses seen amongst adults were: upper respiratory tract infection (URTI, 27.2%), gastritis/gastroenteritis (18.9%), and lower respiratory tract infection (LRTI, 13.8%). For the paediatric group of patients, these were gastritis/gastroenteritis (29.2%), URTI (28.7%), and LRTI (7.5%) [Tables 2 and 3]. 6.3% adults came to seek help for long standing, chronic medical problems i.e. hypertension and diabetes mellitus. Posttraumatic stress disorder/ psychological manifestations were seen in 8.1% of adults and 2.8% of children, whilst injuries and wounds were seen in 10% and 12% of these groups, respectively.

**Table 1:** Daily Patient Load in the Different Villages

Date	Name of the Village	Number of people treated
24 May	Twante	74
	Shwe San Daw	94
	Payangi	87
25 May	Twante	110
	Kun Chan	150
	Payangi	156
26 May	Twante	74
	Kun Chan	214
	Payangi	186
27 May	Twante	80
	Kharloke	256
	Alesu	221
28 May	Twante Wa	356
	Twante	93
	Mi Ba Gane	26
29 May	Twante	106
	Twante Wa	516
30 May	Ka Yun Chaung	639
31 May	Alesu	441
1 June	Tan Yar Gally	581
2 June	Orphanage	250
		4 710 (Total)

**Table 2:** Diagnoses group for adults

Diagnoses	Numbers	% to the nearest 1 decimal point (of adults)
Gatritis/gastroenteritis	551	18.9
Upper respiratory tract infection	795	27.2
Lower respiratory tract infection	403	13.8
Skin problems (scabies, rashes, infection)	141	4.8
Chronic disease (diabetes, hyertension)	183	6.3
Posttraumatic stress disorder/Psychological complaints	238	8.1
Giddiness	132	4.5
Musculo-skeletal pain	187	6.4
Wounds/minor injuries	291	10
Total	2,921	

**Table 3:** Diagnoses group for paediatric patients

Diagnoses	Numbers	Percentage (nearest 1 decimal place)
Gastritis/gastroenteritis	522	29.2
Upper respiratory tract infection	514	28.7
Lower respiratory tract infection	134	7.5
Skin complaints	72	4.0
Posttraumatic stress disorder/Psychological complaints	54	2.8
Giddiness	48	2.7
Headache	80	4.5
Musculo-skeletal pain	88	4.9
Wounds/ Injuries	214	12.0
Cardiovascular	15	0.01
Others	72	2.9
Total	1,789	

There was also a significant proportion of paediatric patients who had nonspecific complaints i.e. giddiness (2.7%), headache (4.5%), and musculoskeletal pain (4.9%).

In the category ‘others’, diagnoses comprised of complaints such as passage of worms per rectal, request for vitamins and health supplements, failure to thrive, convulsions, joint swelling, malaria and request for X-rays or blood tests.

## WRAPPING UP AND RETURN

After the operations, the necessary follow up was planned. This comprised on medium term projects such as water treatment and desalination, rebuilding of schools and dormitories, assistance with emergency and disaster preparedness. The networks established with the local contacts and authorities become very important for subsequent follow up and planning of programmes for execution. Other health related issues which needed handing over were passed on to the local doctors and healthcare staff as well as the international volunteers, such as those from the UN, and UNICEF who would be staying on for a prolonged period.

## DISCUSSION

Tropical cyclones are low pressure weather systems that develop over the warm waters of the ocean typically between latitudes 30 degrees North and South. As warm water is necessary to power cyclone formation, only the mid section of the planet can conceive them. A corollary is that cyclones neither form at nor cross the equator.[9,10]

The public health consequences of cyclones such as CN is tremendous; high mortality rates, widespread loss of clean water, food and supplies, electricity, increased pests and vectors as well as possible toxic exposures.

Disease surveillance have shown that there is an increase number of self-limiting gastrointestinal and respiratory tract illnesses after the cyclone.[9,11,12] These represented the commonest diagnoses Team Singapore managed as well.

Chronic illnesses such as asthma and emphysema have been known to be exacerbated after disasters. However, these were not commonly seen in the population studied in Twante Township.

Injuries, according to some reports, represent the commonest cause of death and the primary cause of morbidity from a tropical cyclone. In fact, the top three causes of cyclone-related injuries are laceration, blunt trauma and puncture wounds, with about 80% of these injuries confined to the lower extremities.[13,14] In this series of nearly 5,000 patients, injuries did not represent the top three diagnoses in both adult and children. There were much less deep wounds as compared to the Asian tsunami, where waves had a much greater force. There could be several reasons for this. Perhaps the majority of those with these injuries, was seriously affected and had contributed to early mortality figures. Also, those with the most injuries would be the population living closest to Ground Zero, at the Delta area. The further away one moves, this trend may be less prominent. It also cannot be denied that some of the patients treated in the remote areas may have taken advantage of having a medical team there to come forward to seek medical treatment. Many have not gotten access to any form of medical treatment or clinic for many years as these were nonexistent.

A WHO report stated there was a great need for psychological support.[3]

In our own series, this was 8.1% and 2.8% in adults and children under 12 years, respectively. Some examples of the presentation included direct complaints of fear and recurring memories of the effects of the cyclone, whilst others presented with headache, giddiness, insomnia, palpitations and a range of other somatization symptoms (bodily aches and pains of no other causes). In these cases, the person may come in and complain of a specific symptom and upon further clarification of the history and examination, there may be no obvious organic diagnoses. Thus, there is the need to probe further into the history. This is where interpreters come in handy especially for foreign medical teams. Amongst the paediatric patients, presentation came in the form of screaming at night and crying, fear of being or sleeping alone (whilst they used to do this before the cyclone), refusal to feed, refusal to go out of the house or to get to school due to fear, not wanting to go near the sea/river and so on. This also depends on the age group of the child. At the orphanage we visited, the majority of the children were psychologically traumatized as there were several deaths from among their friends and there were several ‘new’ children being transferred in as they had lost both their parents to CN.

In counseling these cases, it could be a ‘one to one’ counseling with the presence of an interpreter or, group counseling, such as the one done for the children at the orphanage we visited. The latter was done through the use of drawing therapy, group interactive interviews and simple games. As Team Singapore was only there for two weeks, we empowered the teachers at the orphanage and local volunteers on how to continue with the sessions. There was also a group from UNICEF who was there on a longer term basis who would continue with the counseling and therapy

## LESSONS LEARNT

Preparedness is the key to preserving human health in the event of cyclones and tsunamis. Many may even argue that this is a basic human right. Absence of emergency preparedness will result in death happening immediately during the impact phase, where emergency response activities are most vulnerable and least capable. Preparedness is the aggregate of all the measures and policies taken by humans before an event occurs that reduces the negative impact that would otherwise have been caused by the event. This would comprise of activities such as risk assessment, planning, hazards monitoring, early warning and population protection measures.[9,15] The objectives of preparedness can be viewed to be multi-fold:

- Prevention of morbidity and mortality
- Provision of timely care for victims
- Ensuring restoration of health and health services as soon as possible
- Protection of staff and responders
- Management of adverse climatic and environmental conditions

Preventive and protective measures are the most efficacious actions which can be taken to protect human health from the hazards of cyclones and tsunamis, but they also are the most consistent with the promotion of human dignity and development.

In countries affected by disasters, local government often have to take the initiative to make certain policy changes or implement programmes and training, in alignment with being prepared as a community. For example, the New Zealand government instituted a new policy of disaster response in an attempt to bring about change and development in the relatively remote agricultural region which was struck by a severe cyclone in 1988.[16]

Although the Delta region of Myanmar has experienced previous cyclones, this was the first time it was of such a magnitude and severity. The local authorities and population need to be more proactive in planning a response programme in such an area. There is a need to change the assessment of needs in such situations from a more reactive one to a more anticipatory approach based on systematic data gathered from the last disaster. For example, after the cyclone in Bangladesh in 1991, local authorities looked into the issue of early warning and shelters. When tested out, only 2 of the 5 shelters were usable due to flooding. Thus there is need to relocate these shelters and make them more accessible at the same time. The local authorities also found that women and children must seek shelter at the first warning and the need to be more precise about the time and place of likely impact as well as the recommended course of action. The local communities must be educated about cyclone preparedness and safety.[17,18]

## COLLABORATIVE CULTURE: THE WAY FORWARD

The US Secretary of state, Condoleezza Rice stated that the handling of CN should be a matter of humanitarian crisis and not one of politics.[19] This sentiment was echoed strongly by many around the world as a reaction to the delays by the local government in letting relief supplies and aid in post CN. In contrast with previous large scale disasters (e.g. tsunami of 2004 and South Asian earthquake affecting Pakistan in 2005), where international and regional teams as well as humanitarian aid were welcomed readily into the affected countries, CN in Myanmar took a completely different face with a different approach. With a disaster of such overwhelming scale, a nation's capabilities can be rapidly overcome. That is why regional and international cooperation and goodwill is essential.

Now there is technology and expertise to help predict strong storms approaching, the estimated time before they strike, their track and destructibility. This type of information can be freely shared with countries that lack such resources and technology to track climate changes. Bodies such as the Indian Meteorological Department (IMD) (or others) which have the capabilities and resources as well as a location not too distant from Myanmar can share life saving information in a timely fashion. This can assist nations to activate their emergency and disaster response preparedness plans.

To further reduce the amount of devastation post disaster, coordinated relief helps to alleviate sufferings. After all we are but ‘one humanity under the same blue sky’. This which appeared to be lacking in Myanmar, may have caused many of the post disaster problems, morbidity and mortality we have seen. The World Food Program has stated that Myanmar has less than 10% of the staff, logistics apparatus and general materials that were needed to manage the crisis.[19]

ASEAN (Association of South East Asian Nations) countries responded to CN with a demonstration of regional solidarity through rapid deployment of medical teams from member states, both bilaterally as well as a regional outfit. Once negotiations with Myanmar reached a positive result and teams were allowed entry, no time was wasted. In the midst of the aftermath of CN, ASEAN foreign ministers met in Yangon to discuss regional cooperation and the way forward. There was also an ASEAN Roundtable meeting on 24 June 2008 to renegotiate various issues. It was at these meetings that the Tripartite Core Group (TCG) (ASEAN-United Nations-Government of Myanmar/Ministry of Health) was formed. This subsequently facilitated many activities and operations planned. In fact, following the step-down and debrief, the TCG met up and discussed their experiences, crystallizing best practices for possible future regional mission deployments.

On the part of Team Singapore, following our busy 9 operational days treating 5,000 patients, we also sent in humanitarian aid, which included food rations, blankets, water purification tablets and hygiene packs. We pledged monetary support in the reconstruction projects and are involved in several rebuilding of school projects in the Delta. In the interim period, we supplied special semi-permanent tentage which could be used as temporary school tents and water pumps. On a more medium term basis, we are involved in water filtration projects with desalination capabilities in the Delta. Singapore is also planning to improve the capabilities of the township hospital in Twante and we hope to assist further with disaster preparedness planning with the local community.

## CONCLUSION

Despite the fact that each disaster is different from the previous ones in so many ways, there are always first principles to apply. The response to CN was unique in some ways. In CN, the initial response in the acute phase had to be delayed due to local politics and the execution of work by international humanitarian teams was segmented and segregated to a particular area/township, such that there were no overlaps. The local culture and mindset of the people was also different. During our two week stay, we were given the impression that the locals were very self reliant and resilient. This could have stemmed from the fact that they have always fended for themselves (especially in the remote and village areas) in whatever simple way they know of and thus had never complained much to local authorities.

As humanitarian workers we have to respect local policies and politics and not impose our ideas and practice on others. However, where good and positive influence can be made, changes should certainly be brought about.

## References

[1] Schultz J, Russell J, Espinel Z (2005). Epidemiology of tropical cyclones. Epidemiol Rev.

[2] Boyarsky L, Schneiderman L (2002). Natural and hybrid disasters: Cause, effect and management. Topics Emerg Med.

[3] (2008). Myanmar: WHO Report.

[4] (2008). Cyclone Nargis and the politics of relief and reconstruction: Aid in Burma (Myanmar). JAMA.

[5] (2008). Myanmar Cyclone Nargis: OCHA Situation Report No 34.

[6] World Health Statistics (2007). http://www.who.int/whois/database/core/core_select_process.cfm.

[7] UNICEF At a glance. Myanmar.

[8] http://www.un.org/apps/sg/offthecuff.asp?nid=1164.

[9] Malilay J, Noji EK (1997). Tropical cyclones. In The Public Health Consequences of Disasters.

[10] Keim ME (2006). Cyclone, tsunami and human health. The key role of preparedness. Oceanography.

[11] Lee LE, Fonseca V, Brett KM (1992). Active mortality and surveillance after Hurricane Andrew- Florida. JAMA.

[12] Lim J, Yoon D, Jung G, Joo Kim W, Lee HC (2005). Medical needs of tsunami disaster refugee camps: Experience in South Sri Lanka. Fam Med.

[13] Noji EK (1993). Analysis of medical needs during disasters caused by tropical cyclones: Anticipated Injury Patterns. J Trop Med Hyg.

[14] Meredith JT, Bradley S, Hogan D, Burnstein J Hurricanes. In Disaster Med.

[15] Sundnes K, Birnbaum M (2003). Health Disaster Management: Guidelines for evaluation and research in Utstein Style. Prehospital and Disaster Med.

[16] Parr AR (1994). The efficacy of the neo-liberal individual choice model for encouraging post-disaster change: Developments in the East Cape region of New Zealand following Cyclone Bola (March 1988). Disasters.

[17] Rahman MO, Bennish M (1993). Health related response to natural disasters: The case of the Bangladesh cyclone of 1991. Soc Sci Med.

[18] Bern C, Sniezek J, Mathbor GM, Siddiqi MS, Ronsmans C, Chowdhury AM (1993). Risk factors for mortality in the Bangladesh cyclone of 1991. Bull World Health Organ.

[19] Kyaw NN (2008). The Myanmar Nargis Aftermath: A Disaster in governance. Rajaratnam School of International Studies Commentaries.

